# Dealing with the problem of non-specific *in situ *mRNA hybridization signals associated with plant tissues undergoing programmed cell death

**DOI:** 10.1186/1746-4811-6-7

**Published:** 2010-02-05

**Authors:** Jaana Vuosku, Suvi Sutela, Mira Sääskilahti, Johanna Kestilä, Anne Jokela, Tytti Sarjala, Hely Häggman

**Affiliations:** 1 Department of Biology, University of Oulu, P.O. Box 3000, 90014 Oulu, Finland; 2 Finnish Forest Research Institute, Parkano Research Unit, 39700 Parkano, Finland

## Abstract

**Background:**

*In situ *hybridization is a general molecular method typically used for the localization of mRNA transcripts in plants. The method provides a valuable tool to unravel the connection between gene expression and anatomy, especially in species such as pines which show large genome size and shortage of sequence information.

**Results:**

In the present study, expression of the catalase gene (*CAT*) related to the scavenging of reactive oxygen species (ROS) and the polyamine metabolism related genes, diamine oxidase (*DAO*) and arginine decarboxylase (*ADC*), were localized in developing Scots pine (*Pinus sylvestris *L.) seeds. In addition to specific signals from target mRNAs, the probes continually hybridized non-specifically in the embryo surrounding region (ESR) of the megagametophyte tissue, in the remnants of the degenerated suspensors as well as in the cells of the nucellar layers, i.e. tissues exposed to cell death processes and extensive nucleic acid fragmentation during Scots pine seed development.

**Conclusions:**

In plants, cell death is an integral part of both development and defence, and hence it is a common phenomenon in all stages of the life cycle. Our results suggest that extensive nucleic acid fragmentation during cell death processes can be a considerable source of non-specific signals in traditional *in situ *mRNA hybridization. Thus, the visualization of potential nucleic acid fragmentation simultaneously with the *in situ *mRNA hybridization assay may be necessary to ensure the correct interpretation of the signals in the case of non-specific hybridization of probes in plant tissues.

## Background

The *in situ *hybridization assay that is used for the localization of specific nucleic acid sequences in various organisms, species and specimens is a decades-old technology that is still continuously developed and remains applicable in many modern contexts, such as live-cell imaging [1] and medical diagnostics [2]. In pines, which show both shortage of sequence information and large genome size, *in situ *mRNA hybridization provides a molecular tool for a better understanding of the links between structural components and gene function.

Programmed cell death (PCD) is a fundamental cellular process involved in the selective elimination of misplaced, non-functional or damaged cells. In the life cycle of plants, the first signs of PCD are seen as early as during embryogenesis, when certain cells or even entire tissues or organs die for the sake of correct embryonic pattern formation [3]. In the Scots pine seed, multiple embryos arise from the same zygote, but only the dominant embryo survives and completes the development while subordinate embryos are eliminated via PCD [4]. PCD also causes the deletion of cells in suspensors that serve temporary functions during embryo development [4]. Embryos grow within the corrosion cavity of the megagametophyte, a haploid maternal tissue that can be considered as the functional homolog of endosperm in an angiosperm seed due to its role as a nutrient source of developing embryos [5]. During the Scots pine embryogenesis, the megagametophyte cells in the narrow embryo surrounding region (ESR) are destroyed by sudden necrotic-like cell death to nourish the developing embryo [6,7]. Furthermore, cells of the nucellar layers that surround the outer surface of the megagametophyte die during seed development [6,8].

In a cell, the main target of the PCD machinery is the nucleus, and the degradation processes include both chromatin and nuclear envelope [9]. In PCD, nuclear DNA is fragmented into nucleosomal units that form a ladder on an agarose gel, whereas in necrosis, DNA is degraded randomly and results in a smear [10,11]. *In situ *DNA cleavage can be visualized in individual cells by the TUNEL (terminal deoxyribonucleotidyl transferase (TdT)-mediated deoxyuridine triphosphate (dUTP) nick end labeling) assay [12] as well as on the basis of acridine orange (AO) fluorescence [13], albeit without the possibility to distinguish between internucleosomal and random cleavage [14]. Chromatin degradation is not restricted to the nucleus but may also take place in the cytoplasm [15]. Additionally, in a cell death process with necrotic-like morphology, cells break down and fragmented nucleic acids may be detected also in the surrounding extra-cellular space [6].

In the present study, we show methodology that reveals nucleic acid fragmentation as a reason for non-specific *in situ *mRNA hybridization signal in dying plant tissues. We assessed the *in situ *hybridization of catalase (*CAT*), diamine oxidase (*DAO*) and arginine decarboxylase (*ADC*) mRNA transcripts in developing Scots pine seeds and were faced with non-specific hybridization of probes. In order to understand the reason for the signals that were caused by the sense probes (i.e. revealing the non-specificity of the antisense probes), we included RNase and DNase controls into the *in situ *mRNA hybridization assay treatments, assessed DNA integrity by the AO-staining test and the TUNEL assay and excluded the possibility that RNA probes would have been bound by phenolic compounds or carbohydrates. Our results suggest that extensive nucleic acid fragmentation during cell death processes can be a considerable source of non-specific signals in traditional *in situ *mRNA hybridization.

## Results

### Development of Scots pine seed

In the Scots pine, the development of a mature seed takes two years. In Scandinavia, wind pollination occurs at the beginning of the growing season, usually in late May or early June, after which the pollen tube germination gradually ceases and then continues during the following growing season about one year later [16,17]. The overall embryo development pathway can be divided into three distinct phases, called proembryogeny, early embryogeny and late embryogeny. Proembryogeny includes the stages before the elongation of the suspensor system. Early embryogeny initiates with the elongation of the suspensor system and terminates with the appearance of the root meristem. Late embryogeny culminates in the maturation of the embryo [18]. In the present study, the Scots pine zygotic embryos used for the *in situ *mRNA hybridization assays were at the developmental stage of early embryogeny or had reached the developmental stage of late embryogeny (Additional file 1).

### Nuclear DNA fragmentation in immature Scots pine seed

**Figure 1:**
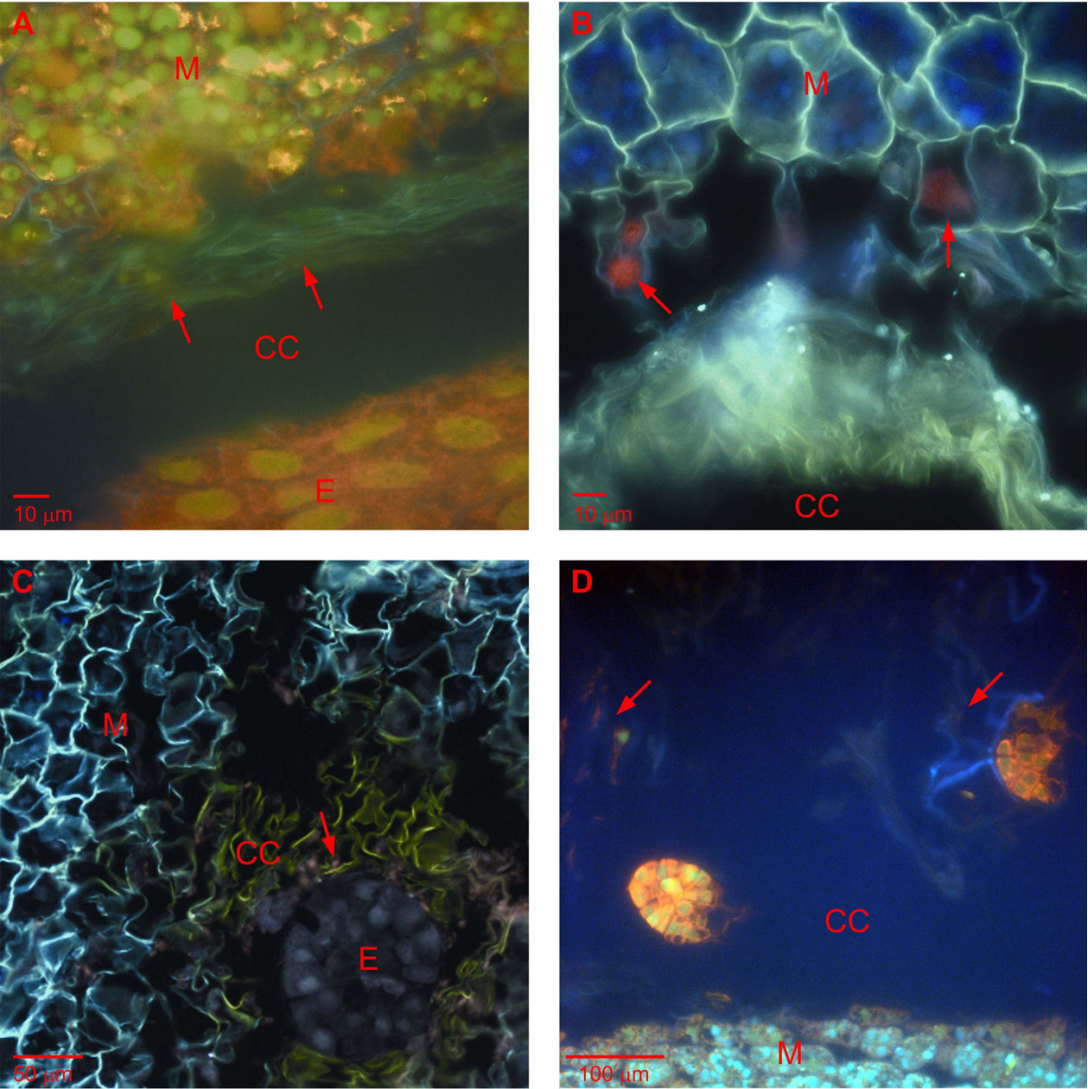
**Nucleic acid fragmentation in immature Scots pine seed**. Nucleic acid fragmentation in the embryo surrounding region (ESR), cells of the megagametophyte tissue and the degenerated suspensor tissue in a developing Scots pine seed. (A) In the acridine orange (AO) -stained section, cell wall remnants and degraded nucleic acid formed a zone between the ESR and the developing embryo (arrows). (B) TUNEL-positive nuclei of the megagametophyte cells in the ESR (arrows). (C) TUNEL-positive signal in fragmented DNA among the cell wall remnants in the corrosion cavity (arrow). (D) AO-stained section with subordinate embryos and fragmented nucleic acids in the corrosion cavity close to the remnants of the degenerated suspensor tissue (arrows). CC = corrosion cavity, E = embryo, M = megagametophyte.

In a pine seed, most of the storage reserves are located in the megagametophyte tissue that surrounds the developing embryos [19]. In an earlier study, we showed that during all the developmental stages of the Scots pine zygotic embryogenesis, the megagametophyte cells in the ESR and in the arrow-shaped zone in front of the dominant embryo die via necrotic-like cell death [6]. Their cell wall, plasma membrane and nuclear envelope broke down with the release of cell debris and nucleic acids into the extra-cellular space of the ESR and, subsequently, to the corrosion cavity. The cell wall remnants and degraded nucleic acid formed a zone between the megagametophyte and the developing embryo, as revealed by the AO-stained section (Figure 1A). The nuclear DNA fragmentation in the dying megagametophyte cells in the ESR and in front of the dominant embryo as well as the fragmented DNA among the cell wall remnants in the corrosion cavity were also revealed with the TUNEL assay (Figure 1B and 1C). In addition to the ESR of the megagametophyte, fragmented nucleic acids were also detected in the corrosion cavity near the remnants of the degenerated suspensor tissue (Figure 1D). The control sections for the AO-staining and TUNEL assays are presented in Additional file 2.

### *In situ *mRNA hybridization signal in fragmented DNA

In the present study, the *in situ *mRNA hybridization assays with antisense and sense probes of catalase (*CAT*), diamine oxidase (*DAO*) and arginine decarboxylase (*ADC*) genes resulted in a uniform non-specific signal in cells with fragmented nucleic acids. Previously, we have discovered a comparable, non-specific signal in *in situ *mRNA hybridization of several genes that are related to polyamine metabolism, such as ornithine decarboxylase (*ODC*), *S*-adenosylmethionine decarboxylase (*SAMDC*), spermidine synthase (*SPDS*) and spermine synthase (*SPMS*) as well as housekeeping gene glyceraldehyde-3-phosphate dehydrogenase (*GAPD*) in developing Scots pine seeds ([20], Vuosku *et al*., unpublished results).

**Figure 2:**
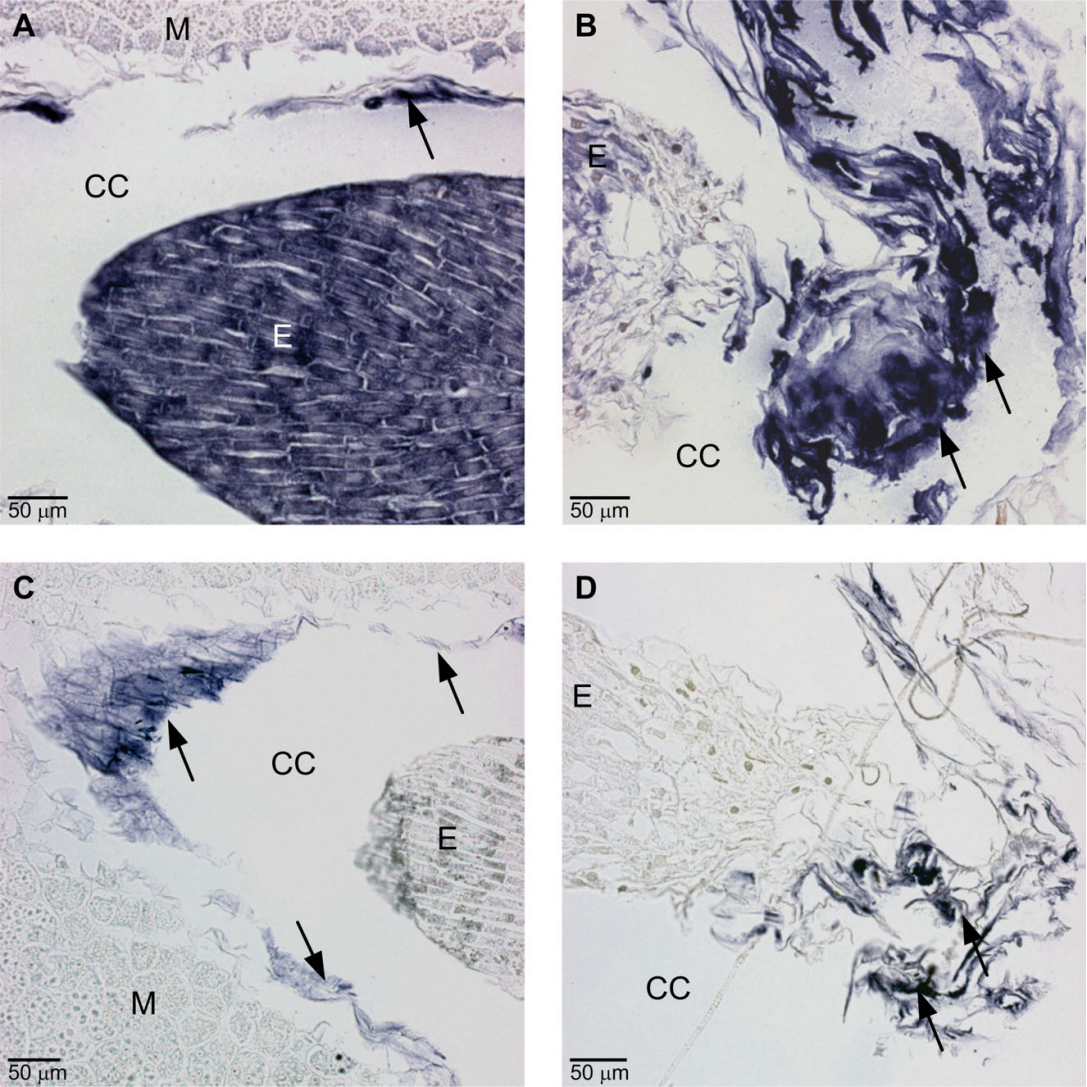
**Catalase (*CAT*) localization by *in situ *mRNA hybridization in immature Scots pine seed**. Localization of catalase (*CAT*) mRNA transcripts by *in situ *mRNA hybridization in a developing Scots pine seed. (A and B) In addition to the specific *in situ *hybridization signal (blue colour) in the embryo, unspecific signals (arrows) were found in the broken megagametophyte cells in the embryo surrounding region (ESR) (A) and in the degenerated suspensor tissue in the corrosion cavity (B) in the section hybridized with the *CAT *antisense probe. (C and D) With the *CAT *sense probe, no signal was found in the embryo but unspecific signals (arrows) were detected in the ESR cells of the megagametophyte and in the arrow-shaped region in front of the expanding corrosion cavity (C) as well as in the degenerated suspensor tissue in the corrosion cavity (D). CC = corrosion cavity, E = embryo, M = megagametophyte.

In the *in situ *mRNA hybridization assays with antisense and sense probes, a non-specific signal was constantly located in the broken megagametophyte cells in the ESR as well as in the cell wall remnants in the corrosion cavity. A non-specific signal was also frequently found in the arrow-shaped region of the megagametophyte tissue, located in front of the expanding corrosion cavity as well as in the degenerated suspensor tissue inside the corrosion cavity (Figure 2; Additional file 3A-C).

No signal was detected in the control sections without a probe or the anti-DIG alkaline phosphatase conjugated antibody (Additional file 4A-E), which confirmed that a non-specific signal did not occur due to interaction between the anti-DIG antibody and dying cells nor due to endogenous alkaline phosphatase activity. By contrast, the non-specific signals remained in the broken megagametophyte cells in the ESR as well as in the zone of cell wall remnants, despite the digestion of RNA (Additional file 4F) or DNA prior to *in situ *hybridization. The TUNEL assay indicated that the DNase treatment created breaks into DNA in the nuclei in the megagametophyte and embryo as well as in the zone consisting of cell wall remnants and degraded nucleic acid in the ESR. Fragmented DNA also leaked from the broken megagametophyte cells in an injury that was probably caused by sample preparation. However, DNA was not completely eradicated by the DNase. Therefore, it was impending that the DNase treatment that took place before *in situ *mRNA hybridization had no reductive effect on the non-specific hybridization of the probes (Additional file 5).

**Figure 3:**
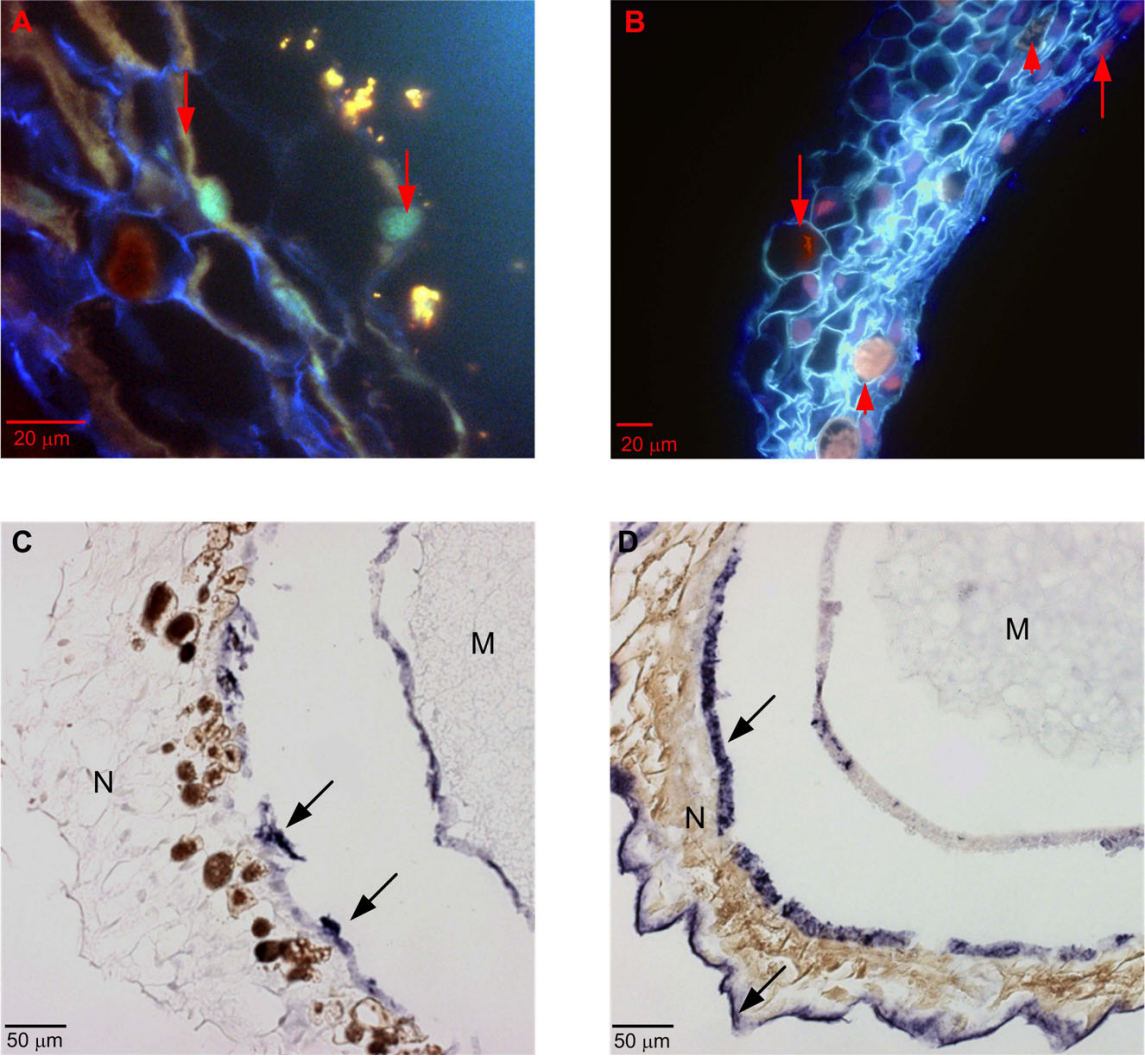
**Nuclear DNA degradation and unspecific *in situ *hybridization signal in immature Scots pine seed**. Nuclear DNA degradation and an unspecific *in situ *hybridization signal in the nucellar layers of a developing Scots pine seed. (A) Fragmented DNA (arrows) in the AO-stained section. (B) TUNEL-positive nuclei (arrows) and autofluorescence of granulous phenols (arrow heads) in the nucellar layers. Localization of diamine oxidase (*DAO*) mRNA transcripts by *in situ *mRNA hybridization (arrows) with antisense (C) and sense (D) probes resulted in equal hybridization signals in the cells of the nucellar layers. M = megagametophyte, N = nucellar layers.

In the Scots pine seed, the nucellar layers located in the cavity between the seed coat and the megaspore membranes (i.e. layers closest to the megagametophyte) surround the megagametophyte [21]. During seed development, cells in the nucellar layers die [6], and in the mature seed, the nucellar layers are composed of compressed cell walls that form an efficient barrier to the passage of water [21]. In the nucellar layers, the cell death process is accompanied by remarkable changes in the morphology of the nuclei and by a huge degradation of nuclear DNA, as revealed by the AO and TUNEL assays (Figure 3A and 3B). The accumulation of phenols in the degenerating cells (Figure 3B) may be a protective mechanism against fungal infections in the mature pine seed [22]. The *in situ *mRNA hybridization with the antisense and sense *DAO *(Figure 3C and 3D) and *ADC *(Additional file 3D and 3E) probes resulted in a strong non-specific signal in the nucellar layers.

The isolation of high-quality RNA from pine tissues is difficult due to phenolic compounds and polysaccharides that may bind RNA [23]. In the present study, however, non-specific *in situ *hybridization signals and granular phenols did not localize in the same area of the nucellar layers, which did not support the potential binding of the RNA probes and phenols (Figure 3). During seed development, starch accumulated into the cells of both the developing embryo and the megagametophyte in which no non-specific *in situ *hybridization signal was found. Thus, the potential cause of the non-specific binding of probes was not connected to polysaccharides (Additional file 6).

## Discussion

*In situ *hybridization has been used in the localization of specific DNA and RNA molecules since the 1960s, and the techniques have undergone continuous evolution during that time [24]. At the beginning, *in situ *hybridization techniques were developed mainly for animal cell cultures or fixed animal tissues, whereas the specific characteristics of plant cells caused several problems and retarded the widespread application of *in situ *mRNA hybridization in plants. In contrast with animal cells, the plant cell wall forms a significant barrier against the penetration of large molecules, such as probes and secondary antibodies, involved in *in situ *hybridization. Furthermore, the high background autofluorescence of chlorophyll and other pigments hinders especially the use of fluorescent probes in photosynthetic tissues [25]. In the present study, we demonstrate that the traditional *in situ *mRNA hybridization technique may be inappropriate for the localization of mRNA transcripts in plant tissues that are exposed to extensive nucleic acid fragmentation during cell death. For the correct interpretation of *in situ *mRNA hybridization results in plant tissues, it is necessary to be aware of the special features of dying plant cells.

The results of the present study strongly suggest that the non-specific binding of the RNA probes in the *in situ *hybridization assay resulted from the hybridization between a probe and fragmented nucleic acid, especially DNA, in the tissues that were exposed to cell death during Scots pine seed development. Although complex gene families may exist in the Scots pine [26], the genomic DNA of a cell still contains only a few copies of the target gene and, therefore, our non-specific *in situ *hybridization results in dying cells cannot be explained by the hybridization between a probe and a genomic target sequence alone. Instead, hybridization might occur between a probe and short oligonucleotides that result from DNA fragmentation by random diffusion events with a few matching base pairs [27].

In plants, PCD may not only occur during embryogenesis, as described with the endosperms of maize [28] and wheat [29] as well as with the suspensors, subordinate embryos [4] and megagametophyte tissue of the Scots pine [6,7], but also during the development of the sporophyte [30-34]. Therefore, the non-specific signaling and/or the misinterpretation of real positives in *in situ *mRNA hybridization may also occur in other plant tissues. Because both specific and non-specific *in situ *hybridization signals may exist in the same plant tissue, a further expression analysis alone is not sufficient to validate the results of *in situ *hybridization. In the case of non-specific hybridization of probes, our results emphasize the importance of nucleic acid degradation assays for revealing potentially incipient or ongoing PCD processes with nucleic acid fragmentation in the plant tissue under investigation.

## Conclusions

During Scots pine seed development, cell death caused extensive nucleic acid fragmentation in the ESR of the megagametophyte tissue, in the remnants of the degenerated suspensors as well as in the cells of the nucellar layers. In traditional *in situ *mRNA hybridization, the sense and antisense probes of several genes were hybridized with fragmented nucleic acids that caused localized non-specific signals. The results emphasize the importance of visualizing potential nucleic acid fragmentation simultaneously with the *in situ *mRNA hybridization assay to ensure the correct interpretation of the signals especially in the case of non-specific binding of probes.

## Methods

### Scots pine immature seeds

One-year-old immature seed cones were collected from an open-pollinated elite Scots pine (*Pinus sylvestris *L.) clone K818 in Punkaharju, Finland (61°48' N; 29°17' E) during one growing season. The collection was repeated four times in July throughout the period of embryo development as described in detail in [6]. Immature seeds were dissected from developing cones and fixed for anatomical and histochemical studies, nucleic acid degradation assays and an *in situ *mRNA hybridization assay as described below.

### Preparation of specimens for microscopical studies

The immature seeds were fixed immediately in 4% (w/v) p-formaldehyde in 1× PBS buffer (10 mM phosphate, 150 mM NaCl, pH 7.4). After gradual dehydration, ethanol was replaced first by tertiary butanol and then gradually by paraffin. Sections (7 μm) were cut from the embedded samples with a microtome, mounted on SuperFrost^®^Plus slides (Menzel-Gläser) and fixed by drying overnight at 37°C. The paraffin sections were dewaxed in Histochoice (Sigma) and rehydrated through a graded series of ethanol for all microscopical studies.

### Anatomical and histochemical observations

For studying the developmental stage of the zygotic embryos, the sections were stained with toluidine blue (0.05% toluidine blue in H_2_O), and for the detection of starch, the sections were stained with 0.5% potassium iodide-iodine (IKI) [35]. The sections were studied with a light microscope (Nikon Eclipse E600) and photographed with a Qimaging Micropublisher 5.0 RTV digital camera. Adobe Photoshop CS was used to adjust contrast, brightness and colour uniformly in entire images for all studies.

### *In situ *detection of DNA fragmentation

For the observation of nucleic acids and the evaluation of chromatin stability, the sections were stained by a dual fluorescence dye, acridine orange (AO) (1.6 mM), according to Bouranis *et al*. [36]. In the AO-stained sections, the double stranded nucleic acid (i.e. DNA) fluoresces green and the single stranded (i.e. RNA) fluoresces red. Fragmented DNA emits fluorescence in a spectrum varying from yellow-green to red [13].

The TUNEL assay was used for the *in situ *detection of DNA strand breaks. The sections were digested with 10 μg mL^-1 ^proteinase-K (Roche Molecular Biochemicals) for 30 min and washed two times with PBS buffer (10 mM phosphate, 150 mM NaCl, pH 7.4), after which the sections were labelled with the TMR red (red fluorescence) *in situ *cell death detection kit (Roche Molecular Biochemicals) according to the manufacturer's protocol. A label solution without terminal transferase instead of the TUNEL reaction mixture was used for the negative control. The TUNEL-stained sections as well as the AO-stained sections were examined under a microscope (Laser Scanning Microscope LSM 5 PASCAL, Carl Zeiss), using an HBO 103 mercury lamp.

### *In situ *mRNA hybridization analysis

The RNA antisense and sense probes for the *in situ *localization of catalase (*CAT*), diamine oxidase (*DAO*) and arginine decarboxylase (*ADC*) mRNA transcripts were prepared by a PCR-based technique in which a T7 polymerase promoter sequence (TAATACGACTCACTATAGGG) was introduced at the 5' ends of the gene-specific primers [37]. The PCR primers for the preparation of the *ADC *antisense and sense probes were presented in an earlier paper of ours [20], and the PCR primers for the *CAT *probes were 5'-AACCACAGTCATGCAACCAA-3' and 5'AGACCAGGACCAAATGCAAG-3' and for the *DAO *probes 5'-ATTTCAGGCATGGAGATTCG-3' and 5'-ATTCTTCACCGTTTGCTTGG-3'.

PCR fragments were produced under standard PCR conditions using DyNazyme™EXT polymerase (Finnzymes) and plasmid DNA that contained the cDNA in question as the template. The PCR fragments were gel-purified with the DNA Gel Extraction Kit (Millipore Corporation), and 250 ng was subsequently used as a template DNA for *in vitro *transcription by T7 RNA polymerase (Invitrogen), incorporating dig-UTP via DIG RNA labelling Mix (Roche Molecular Biochemicals). The template DNA was digested with 2 U of amplification grade DNase (Invitrogen) in a reaction volume of 20 μL for 15 min at room temperature, and the probe was purified with the NucleoSpin^® ^RNA Clean-Up kit (Macherey-Nagel). The lengths of the *CAT *and *DAO *probes were 245 and 342 nucleotides, respectively. After RNA transcription, the length of the *ADC *probe was 566 nucleotides, but the *ADC *probe was hydrolyzed in 1× carbonate buffer (80 mM NaHCO3, 120 mM Na2CO3) at 60°C for 15 min after which the length was 250-300 nucleotides.

In the *in situ *mRNA hybridization procedure that was used, the dewaxed sections were treated sequentially with 0.2 M HCl, proteinase K (10 μg mL^-1^), 4% (w/v) p-formaldehyde and 0.5% acetic anhydride in 0.1 M triethanolamine. The samples were hybridized in a solution containing 50% (v/v) formamide, 300 mM NaCl, 10 mM Tris (pH 7.0), 10 mM Na3PO4 (pH 7.0), 50 mM EDTA, 10% dextran sulphate, 200 μg mL^-1 ^tRNA, 1× Denhardt's solution and 10 U mL^-1 ^RNase inhibitor overnight at 55°C in a water atmosphere. The amount of RNA probe used was about 200 ng per slide. After hybridization, the slides were washed in 0.2× SSC buffer (30 mM NaCl, 3 mM sodium citrate, pH 7.0) at 55°C for 60 min and treated with DNase-free RNase A (10 μg mL^-1^) in NTE buffer (500 mM NaCl, 10 mM Tris (pH 8.0), 5 mM EDTA). The hybridized probes were detected using an alkaline phosphatase-conjugated anti-DIG antibody and NBT/BCIP as substrates (blue colour) (Roche Molecular Biochemicals).

In all the *in situ *hybridization experiments, the following positive and negative controls were used. An adjacent tissue (i.e. Scots pine zygotic embryo) known to contain *CAT, DAO *and *ADC *mRNA transcripts was used as positive control. In addition to hybridization using a sense probe (a sequence identical but not complementary to the target sequence), digestion of RNA with RNase A (50 μg mL^-1 ^in NTE buffer at 37°C for 30 min) and DNA with DNase (3 U/μl) prior to *in situ *hybridization, hybridization without a probe as well as detection of a probe without the anti-DIG antibody were used as negative controls.

## List of abbreviations

ADC: arginine decarboxylase; AO: acridine orange; CAT: catalase; DAO: diamine oxidase; ESR: embryo surrounding region; GAPD: glyceraldehyde-3-phosphate dehydrogenase; ODC: ornithine decarboxylas; PCD: programmed cell death; SAMDC: *S*-adenosylmethionine decarboxylase; SPDS: spermidine synthase; SPMS: spermine synthase; TUNEL: terminal deoxyribonucleotidyl transferase (TdT)-mediated deoxyuridine triphosphate (dUTP) nick end labeling.

## Competing interests

The authors declare that they have no competing interests.

## Authors' contributions

JV designed the study with HH; carried out the analyses with SS, MS, JK, AJ and TS and wrote the manuscript with SS, AJ, TS and HH. All the authors have read the manuscript and agree with the content.

## Supplementary Material

Additional file 1 **Developmental stages of Scots pine zygotic embryos**. (A) The dominant embryo and subordinate embryos in the corrosion cavity surrounded by the embryo surrounding region (ESR) of the megagametophyte at the developmental stage of early embryogeny. (B) The dominant embryo in the corrosion cavity at the developmental stage of late embryogeny. The megagametophyte is surrounded by the nucellar layers, and the ESR is characterized by necrotically dying cells. CC = corrosion cavity, E = embryo, ESR = embryo surrounding region, M = megagametophyte, NL = nucellar layers, SE = subordinate embryo. Click here for file

Additional file 2 **Negative controls for AO and TUNEL assays**. Immature Scots pine seed in the developmental stage of late embryogeny. (A) Control sample with no AO staining. (B) Negative control for the TUNEL assay (omission of TdT). CC = corrosion cavity, E = embryo, M = megagametophyte. Click here for file

Additional file 3 **Localization of arginine decarboxylase (*ADC*) mRNA transcripts by *in situ *mRNA hybridization in developing Scots pine seed**. Localization of arginine decarboxylase (*ADC*) mRNA transcripts by *in situ *mRNA hybridization. (A) In the section hybridized with the *ADC *sense probe, the signal (blue colour) was found in the embryo surrounding region (ESR) cells of the megagametophyte and in the arrow-shaped region (arrows) in front of the expanding corrosion cavity as well as in the suspensor tissue (double arrow). (B and C) With the *ADC *antisense probe, unspecific signals were detected in the arrow-shaped region of the megagametophyte tissue in front of the expanding corrosion cavity (B) as well as in the degenerated suspensors in the corrosion cavity (C). (D and E) Non-specific signals in the nucellar layers in sections hybridized with the antisense (D) and sense (E) *ADC *probes. (F) Positive control, the *in situ *hybridization signal in the dividing cells of an embryo hybridized with the antisense *ADC *probe. (G) Labeling with the sense *ADC *probe indicating no signal in the dividing embryo cells. CC = corrosion cavity, E = embryo, M = megagametophyte. Click here for file

Additional file 4 **Controls for mRNA *in situ *hybridization assays**. (A and B) Sections without a probe. (C, D and E) Sections in which the detection of the *CAT *antisense probe was performed without an anti-DIG alkaline phosphatase conjugated antibody. (F) Non-specific *in situ *hybridization signal (blue colour) in the section treated with RNase A before hybridization with the *ADC *antisense probe. CC = corrosion cavity, E = embryo, M = megagametophyte, N = nucellar layers. Click here for file

Additional file 5 **DNase treated controls for TUNEL and *in situ *mRNA hybridization assay**. DNase treatment created DNA breaks in a developing Scots pine seed. (A and B) TUNEL-stained sections of DNase treatment created DNA breaks in the nuclei, in the zone consisting of cell wall remnants and degraded nucleic acid (arrows) in the ESR and in the injured megagametophyte cells. (C) A positive *in situ *hybridization signal in the ESR (blue colour) in a DNase treated control. CC = corrosion cavity, E = embryo, ESR = embryo surrounding region, M = megagametophyte, SE = subordinate embryo. Click here for file

Additional file 6 **Different localization of starch and non-specific *in situ *hybridization signal**. A non-specific *in situ *hybridization signal and starch were localized to different tissues of a developing Scots pine seed. (A) Histochemical localization of starch grains by potassium iodide-iodine in the embryo and megagametophyte tissue at the late embryogeny stage. (B) Non-specific hybridization of the sense probe of *DAO *(blue colour) in the ESR of the megagametophyte tissue at the late embryogeny stage. CC = corrosion cavity, E = embryo, ESR = embryo surrounding region, M = megagametophyte, NL = nucellar layers. Click here for file
