# Supplementary figures and images for: Dealing with the problem of non-specific in situ mRNA hybridization signals associated with plant tissues undergoing programmed cell death
Source: Plant Methods. 2010 Feb 5;6:7. doi: 10.1186/1746-4811-6-7 (PMC2829549; doi:10.1186/1746-4811-6-7)

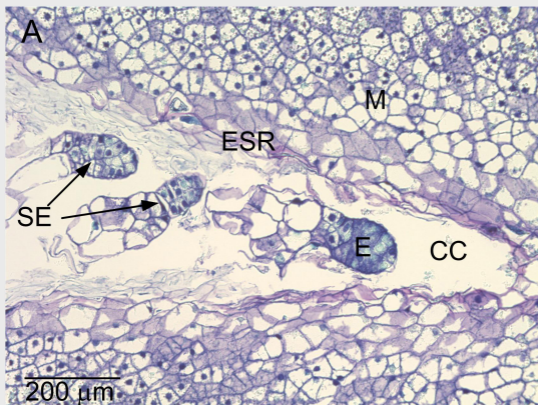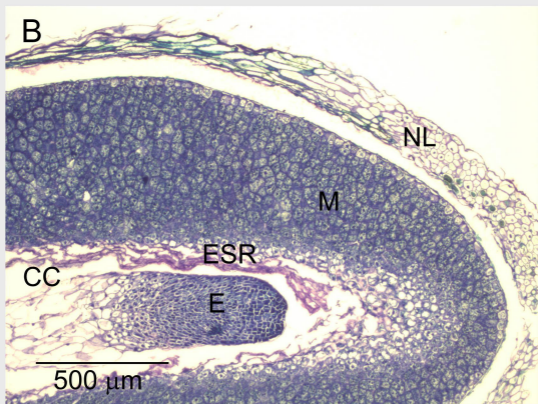

Supplement: Additional file 1 — Developmental stages of Scots pine zygotic embryos. (A) The dominant embryo and subordinate embryos in the corrosion cavity surrounded by the embryo surrounding region (ESR) of the megagametophyte at the developmental stage of early embryogeny. (B) The dominant embryo in the corrosion cavity at the developmental stage of late embryogeny. The megagametophyte is surrounded by the nucellar layers, and the ESR is characterized by necrotically dying cells. CC = corrosion cavity, E = embryo, ESR = embryo surrounding region, M = megagametophyte, NL = nucellar layers, SE = subordinate embryo. [file 1746-4811-6-7-S1.PDF]

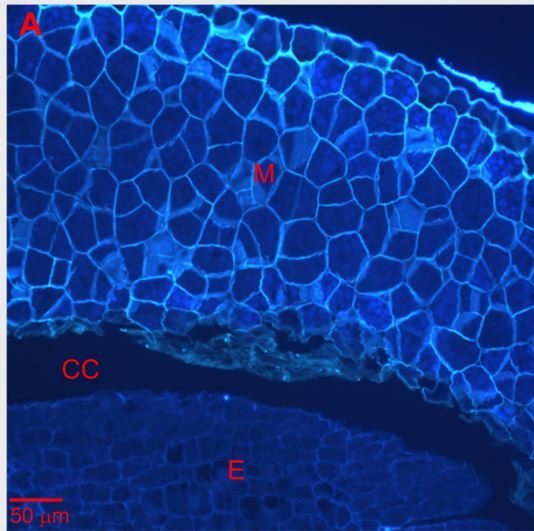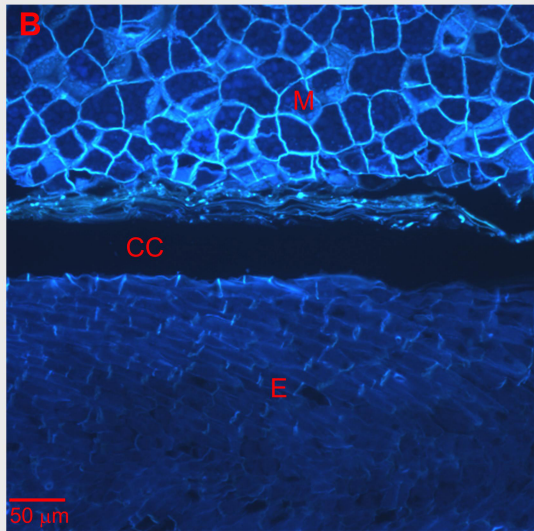

Supplement: Additional file 2 — Negative controls for AO and TUNEL assays. Immature Scots pine seed in the developmental stage of late embryogeny. (A) Control sample with no AO staining. (B) Negative control for the TUNEL assay (omission of TdT). CC = corrosion cavity, E = embryo, M = megagametophyte. [file 1746-4811-6-7-S2.PDF]

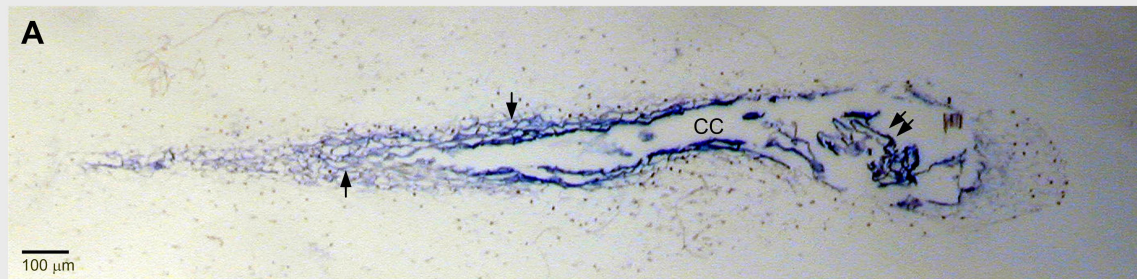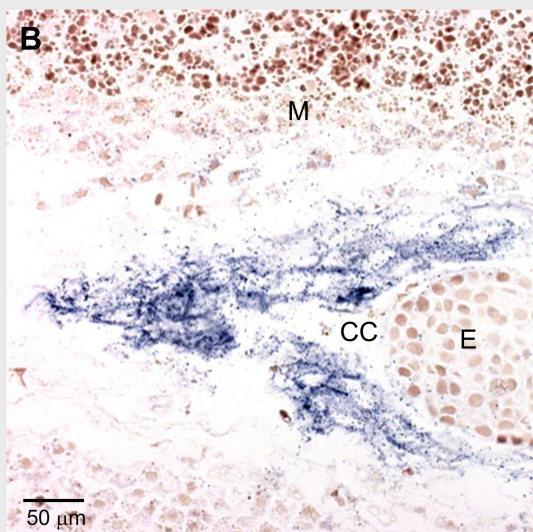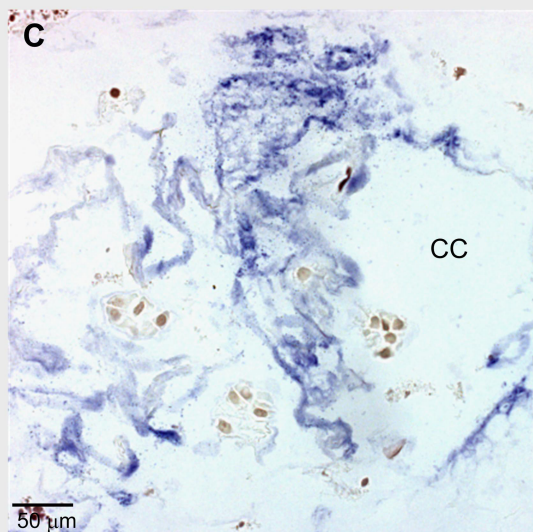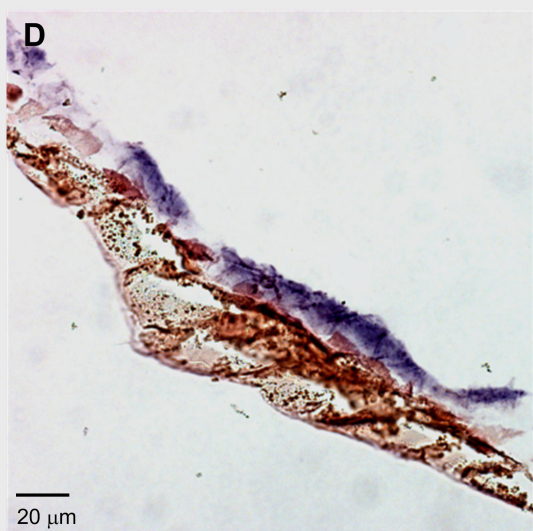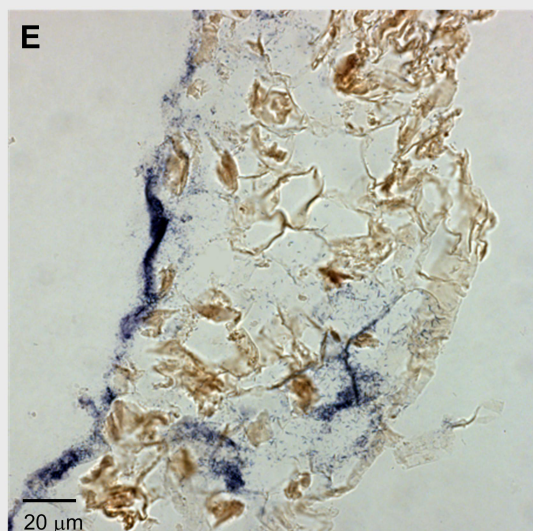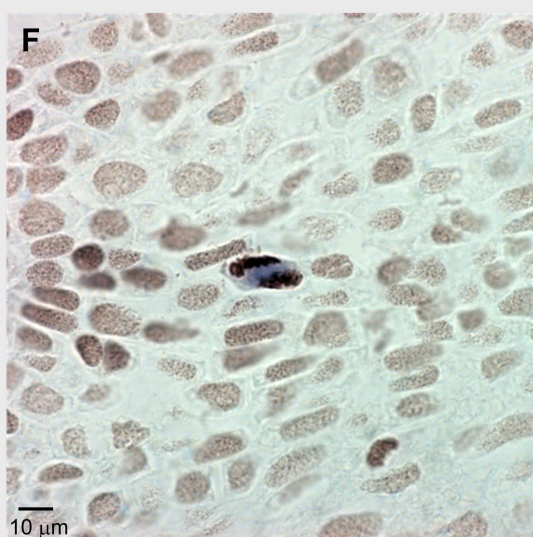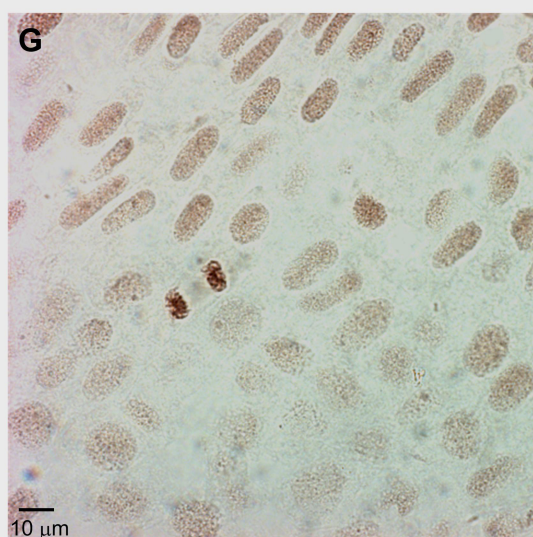

Supplement: Additional file 3 — Localization of arginine decarboxylase (ADC) mRNA transcripts by in situ mRNA hybridization in developing Scots pine seed. Localization of arginine decarboxylase (ADC) mRNA transcripts by in situ mRNA hybridization. (A) In the section hybridized with the ADC sense probe, the signal (blue colour) was found in the embryo surrounding region (ESR) cells of the megagametophyte and in the arrow-shaped region (arrows) in front of the expanding corrosion cavity as well as in the suspensor tissue (double arrow). (B and C) With the ADC antisense probe, unspecific signals were detected in the arrow-shaped region of the megagametophyte tissue in front of the expanding corrosion cavity (B) as well as in the degenerated suspensors in the corrosion cavity (C). (D and E) Non-specific signals in the nucellar layers in sections hybridized with the antisense (D) and sense (E) ADC probes. (F) Positive control, the in situ hybridization signal in the dividing cells of an embryo hybridized with the antisense ADC probe. (G) Labeling with the sense ADC probe indicating no signal in the dividing embryo cells. CC = corrosion cavity, E = embryo, M = megagametophyte. [file 1746-4811-6-7-S3.PDF]

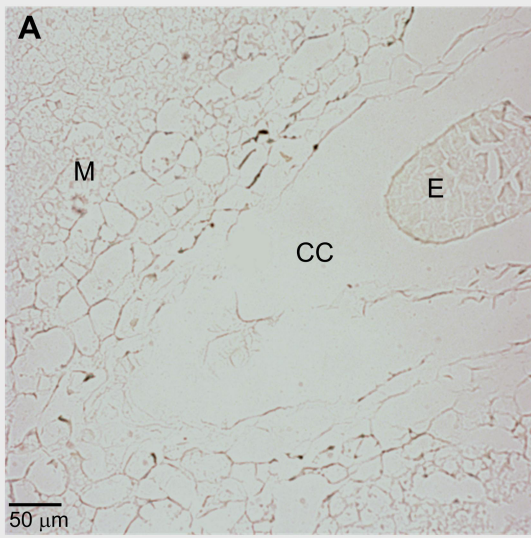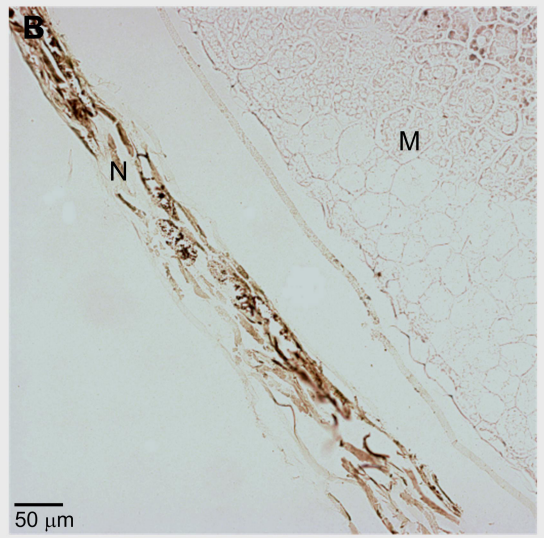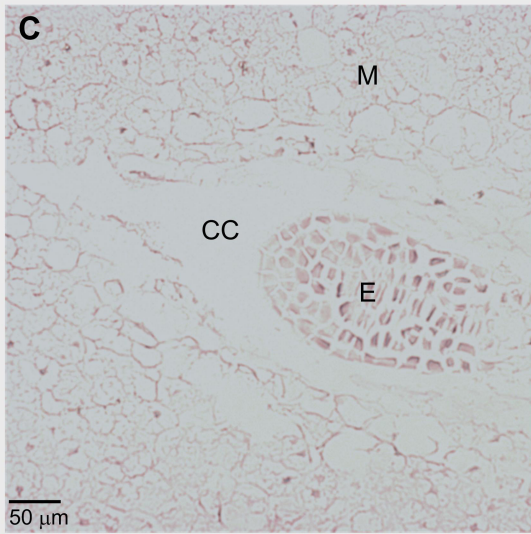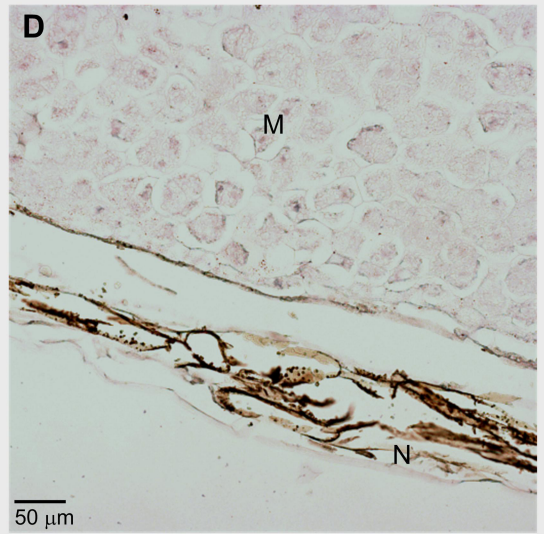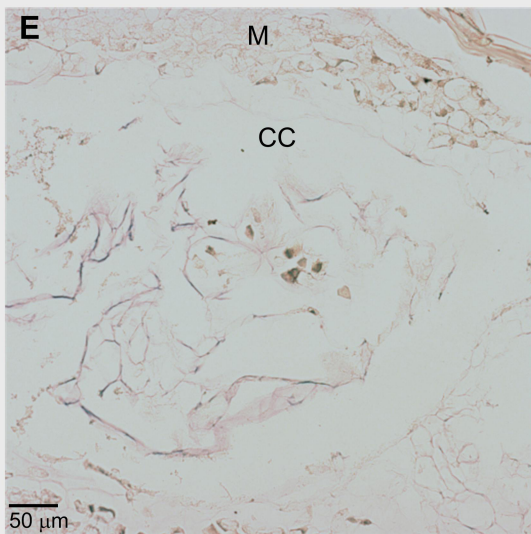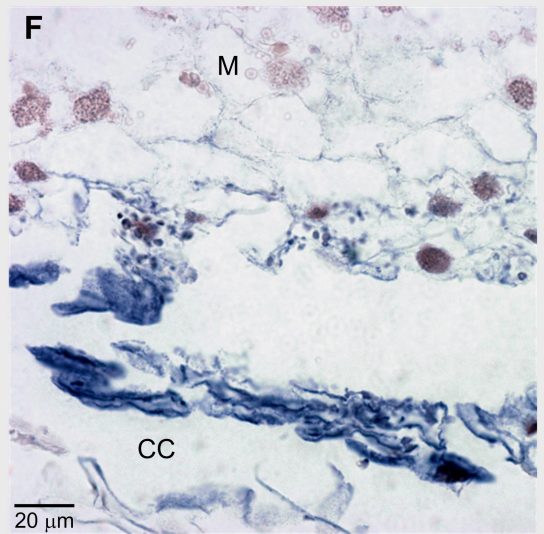

Supplement: Additional file 4 — Controls for mRNA in situ hybridization assays. (A and B) Sections without a probe. (C, D and E) Sections in which the detection of the CAT antisense probe was performed without an anti-DIG alkaline phosphatase conjugated antibody. (F) Non-specific in situ hybridization signal (blue colour) in the section treated with RNase A before hybridization with the ADC antisense probe. CC = corrosion cavity, E = embryo, M = megagametophyte, N = nucellar layers. [file 1746-4811-6-7-S4.PDF]

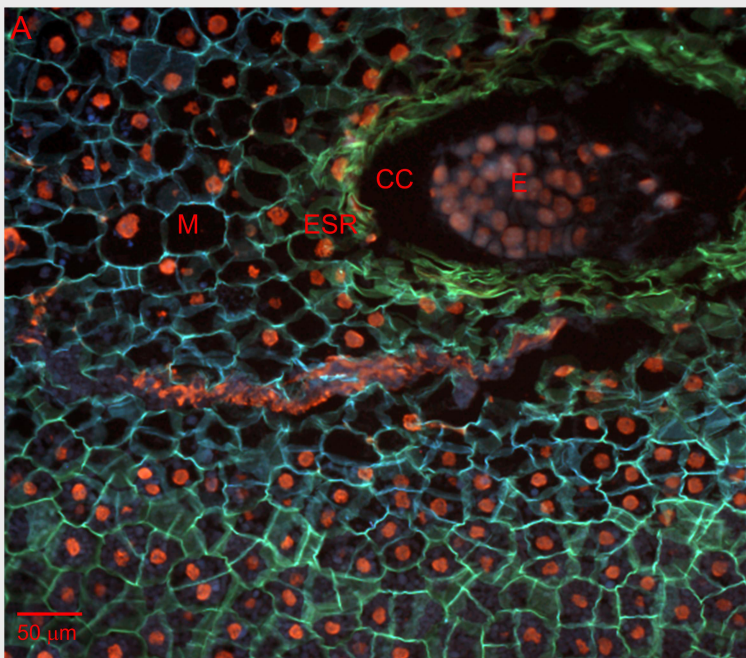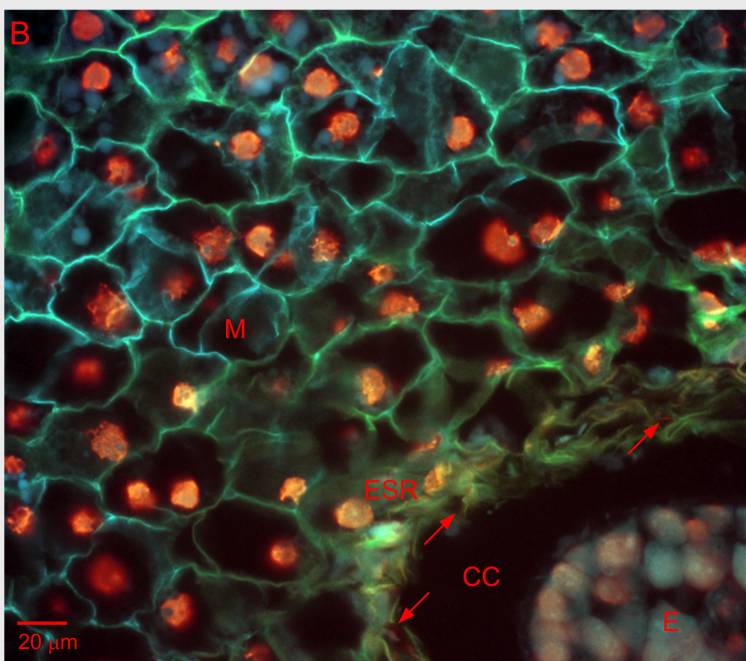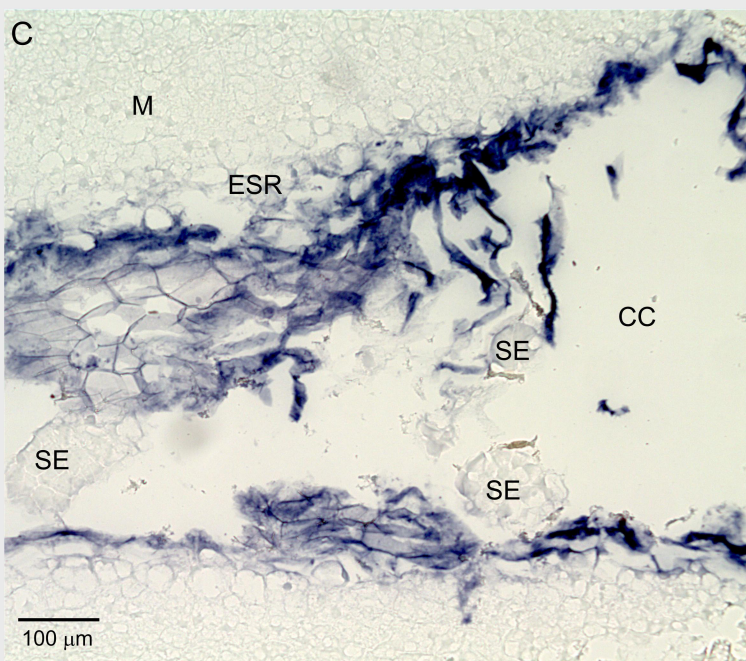

Supplement: Additional file 5 — DNase treated controls for TUNEL and in situ mRNA hybridization assay. DNase treatment created DNA breaks in a developing Scots pine seed. (A and B) TUNEL-stained sections of DNase treatment created DNA breaks in the nuclei, in the zone consisting of cell wall remnants and degraded nucleic acid (arrows) in the ESR and in the injured megagametophyte cells. (C) A positive in situ hybridization signal in the ESR (blue colour) in a DNase treated control. CC = corrosion cavity, E = embryo, ESR = embryo surrounding region, M = megagametophyte, SE = subordinate embryo. [file 1746-4811-6-7-S5.PDF]

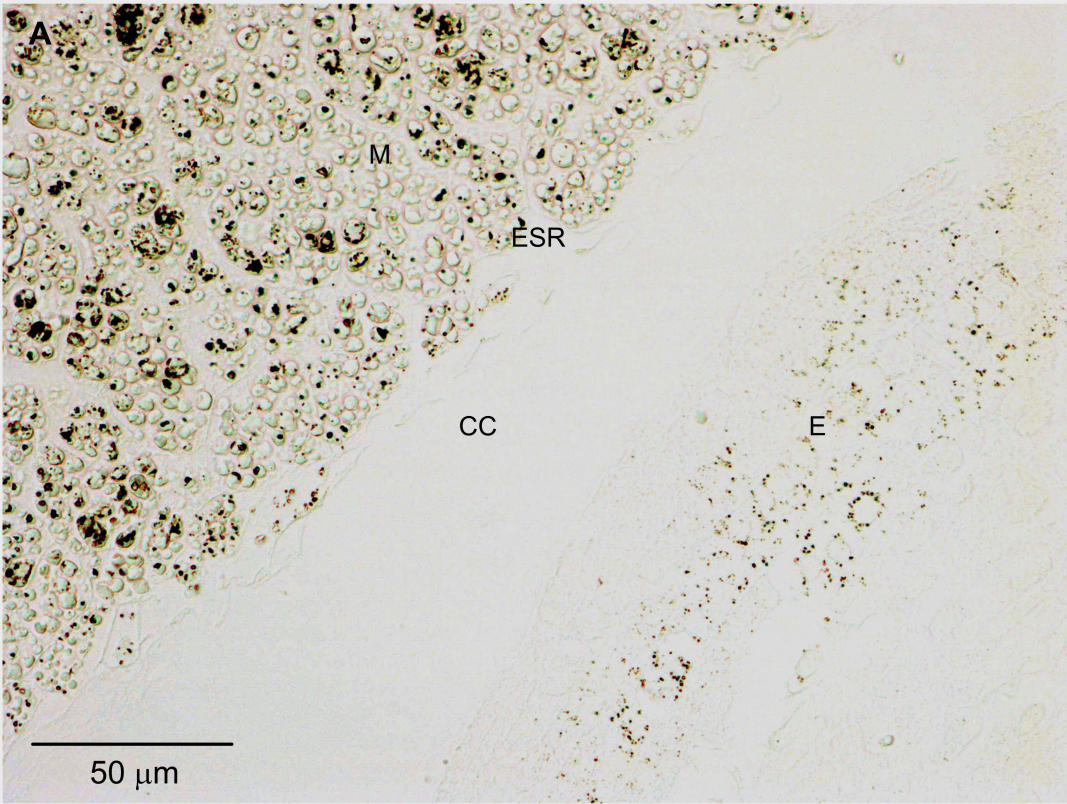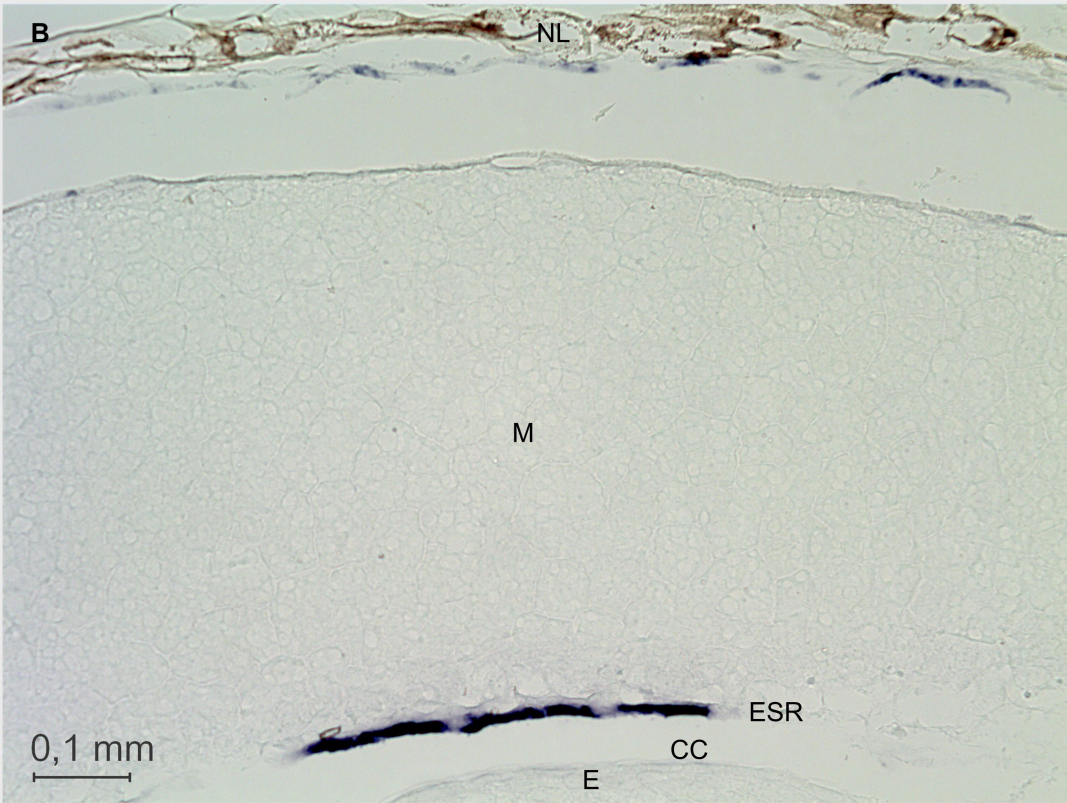

Supplement: Additional file 6 — Different localization of starch and non-specific in situ hybridization signal. A non-specific in situ hybridization signal and starch were localized to different tissues of a developing Scots pine seed. (A) Histochemical localization of starch grains by potassium iodide-iodine in the embryo and megagametophyte tissue at the late embryogeny stage. (B) Non-specific hybridization of the sense probe of DAO (blue colour) in the ESR of the megagametophyte tissue at the late embryogeny stage. CC = corrosion cavity, E = embryo, ESR = embryo surrounding region, M = megagametophyte, NL = nucellar layers. [file 1746-4811-6-7-S6.PDF]
